# Another failure to wean away from fossil fuels: Can we risk it?

**DOI:** 10.1016/j.lansea.2022.100126

**Published:** 2022-12-05

**Authors:** The Lancet Regional Health – Southeast Asia

The 27th UN Climate Change Conference or Conference of the Parties of the UN Framework Convention on Climate Change (COP27) in Egypt was concluded on November 18, 2022, with world leaders yet again failing to confirm commitments to phase out fossil fuels in the near future. Although the Parties scrambled to reach a consensus on loss and damage funding and climate finance, exactly how these promises will be delivered remains uncertain. “We are on a highway to climate hell with our foot still on accelerator,” the UN Secretary General Antonio Guterres opened COP27 with an address to world leaders to act now on climate change before it is irreversible. WHO called upon governments to phase out fossil fuels and transition to clean energy by creation of a fossil fuel non-proliferation treaty, which would ensure that the phase out is fair and equitable. Yet the overrun COP27 closed with delegates exhausted and fossil fuel progress stuck in slow gear.

In southeast Asia, emission-induced climate change is driving floods, landslides, droughts, and sea level to rise, worsening the current health issues of malnutrition, and diarrhoeal and infectious disease. In this issue of *The Lancet Regional Health – Southeast Asia*, Sajith Kumar and colleagues examine air pollution-attributed disease burden across India and warn against using economic productivity as an excuse for continued air pollution and the subsequent health impacts in the country. Further discussion by Bhishma Tyagi and Sachin and Gargi Sarode unpack the debate on how economic development perpetuates more dependence on fossil fuels. Authors argue that least economically developed regions face similar health risk by burning fossil fuels for cooking and transport.

In the seventh Lancet Countdown on health and climate change report, which provides an independent assessment of progress towards the goals of the Paris agreement, more than 90 researchers urged world leaders to take affirmative action of reducing fossil fuels and subsidising greener renewable sources of fuel. They loathe the arrogance of energy giants who are earning record profits, ignoring human health, and taking advantage of COP conferences as a venue to sign side deals.

Southeast Asian economies committed to reducing carbon emissions at COP26 Glasgow in 2021. Sri Lanka set an ambitious target of increasing renewable energy generation from 35% to 70% by 2030 with its “Climate Prosperity Plan”. Nepal has set goals for carbon-neutral tourist destinations by 2030. Indonesia, a country which is 60% powered by coal; plans to achieve net zero carbon emissions by 2060. India committed to reaching carbon neutrality by 2070 and drawing half of its energy requirement from renewable sources by 2030. However, India's public sector units continue to make huge investments in the fossil fuel energy sector. According to a recent study, government subsidies to the renewable sector in India have fallen by nearly 45% whereas oil and gas subsidies increased by around 16% in 2020. In 2021, environmental regulations were pushed back to increase domestic coal production. The government suspended cooking gas subsidies, but is yet to provide alternate support for clean cooking. India has also ambitiously established a solar home power systems programme particularly in rural areas of South India that are still not covered by the electricity grid. However, the benefits have not been extended to the rural masses due to prohibitive costs and dubious financing mechanisms, which is one of the major reasons for shortage of basic medical care in rural areas.

This transition will evidently require substantial large scale financial investments, not only to reduce emissions but also to mitigate climate change effects leading to loss of life and livelihoods in most vulnerable countries. An equitable transition across all sectors is required, which includes changes in policies and regulations, infrastructure, alternative options and livelihood opportunities, and social protection for communities. In India, subsidies for electric vehicles have risen through subsidies for manufacturers and consumers and charging infrastructure to ensure the production of cheaper and more efficient electric vehicles to tackle the current low demand. India has committed to conversion to electric vehicles by 2030 and incentivised electric vehicles with tax breaks, but these are not enough for a fair and equitable transition.

Confronting the issue of climate change offers an opportunity toward developing a healthy ecosystem that helps us move towards a sustainable, climate-resilient, and low-carbon economy that puts health first. Climate change is expected to kill an additional 250,000 people per year from malnutrition, malaria, diarrhoea, and heat stress. Southeast Asia presents as one the most vulnerable regions to the worsening impacts of climate on people's health. In Glasgow, countries had agreed to phase-down fossil fuel use, but oil and gas rich nations managed to derail the progress this year, which speaks poorly about our commitment. COP27 was yet another missed opportunity to arrest climate change.

